# Collagen I and III in women with diastasis recti

**DOI:** 10.6061/clinics/2018/e319

**Published:** 2018-05-28

**Authors:** Rosa Maria Blotta, Sirlei dos Santos Costa, Eduardo Neubarth Trindade, Luise Meurer, Manoel Roberto Maciel-Trindade

**Affiliations:** I Cirurgia, Faculdade de Medicina, Universidade Federal do Rio Grande do Sul (UFGRS), Porto Alegre, RS, BR; II Unidade de Patologia Experimental, Hospital de Clinicas de Porto Alegre (HCPA), Porto Alegre, RS, BR; III Servico de Cirurgia Digestiva, Hospital de Clinicas de Porto Alegre (HCPA), Porto Alegre, RS, BR

**Keywords:** Abdomina Wall, Linea Alba, Diastasis Recti, Type I Collagen, Type III Collagen

## Abstract

**OBJECTIVES:**

Interest in elucidating the etiology of hernias has encouraged countless studies of musculoaponeurotic structures in individuals with and without hernias. Studies of hernia patients have firmly demonstrated a correlation between hernias and collagen alterations in their fascia. Diastasis recti is an increased width of the abdominal midline that is exclusively composed of interlacing aponeurotic expansions of the anterolateral abdominal muscles. The condition is common among women undergoing abdominoplasty, and many factors, not only mechanical, play a role. The goal of this study is to evaluate and compare collagen type I and III levels in the midline fascia of women with and without diastasis recti to report their possible influence on this condition.

**METHODS:**

This is a case-control study nested within a surgical cohort of 18 women with diastasis recti and 18 women without the condition (cases and controls, respectively). Fascia from the midline of the abdominal wall was collected and analyzed through immunohistochemistry using polyclonal antibodies to collagen type I and III.

**RESULTS:**

Both type I and type III collagen were less abundant in women with diastasis recti than in those without the condition, and the difference was statistically significant (*p*<0.001).

**CONCLUSION:**

Low collagen type I and type III levels in the midline of the abdominal wall may play a key role in the development of diastasis recti.

## INTRODUCTION

The anterior abdominal wall and its structures have been the subject of countless studies. The midline, which comprises interlacing aponeurotic expansions of abdominal anterolateral muscles, is easily identified and visualized during the classic abdominal dermolipectomy. The surgery enables observation of the broad variation between the anatomy of the linea alba and abdominal protrusion in these patients.

The myoaponeurotic layer of the anterior abdominal wall plays a key role, both functionally and aesthetically. The linea alba, region of the abdominal wall comprised of aponeurosis and without muscular covering, is exposed to the full intra-abdominal pressure, and its resistance is a crucial factor in the abdominal contention.

To understand factors that may explain failure of abdominal wall correction and containment, some studies note that deformities in the myoaponeurotic layer of the anterior abdominal wall may be caused by changes in type I and III collagen in the fascia of these structures 1,2.

The fibrillary collagen types I and III are the main components of the interstitial matrix. These collagens are important for tissue stability and function 3.

As a constituent of the abdominal fascia and aponeuroses, including the linea alba, collagen has the important structural role of providing support and resistance to the abdominal wall against intra-abdominal pressure. Type I collagen is the most abundant form; it is the main component of aponeuroses, tendons, and mature scar tissue, and its main function is to provide resistance to tensile stress. Type III collagen corresponds to what was classically described as reticular fiber, and its function is to provide support to expandable structures. Type III collagen levels also increase during the early stages of wound repair 4.

Studies have shown that patients with hernias of various etiologies have lower type I and III collagen levels than control cadavers with no history of hernia 4, highlighting the importance of collagen in the body’s support structures.

An analysis of collagen I and III gene expression in the fascia of obese patients showed that expression levels of type I and III collagens were decreased in obese patients compared with those in non-obese control individuals 5.

On the basis of this evidence, the present study sought to quantify type I and III collagen in the linea alba of abdominal dermolipectomy patients with or without diastasis recti to ascertain whether a difference exists in the collagenous makeup of this anatomical structure in the absence or presence of diastasis.

## PATIENTS AND METHODS

This nested case-control study was carried out within a cohort of patients with indication for abdominal dermolipectomy, including a group of female patients with diastasis recti and a control group of women without this condition. Type I and III collagen were quantitated in samples obtained from supra- and infraumbilical sites along the linea alba in both groups.

The case group comprised female patients between the ages of 30 and 45 years who underwent classic abdominal dermolipectomy and were diagnosed with diastasis recti according to the Rath criteria 6.

In a study of the linea alba from 1996, Rath et al. 6 proposed two definitions of diastasis recti depending on the age of the patient: in patients aged 45 years or younger, diastasis is defined as a separation of the rectus abdominis muscles of more than 10 mm (if above the umbilicus), 27 mm (at the level of the umbilicus), or 9 mm (below the umbilicus); in patients older than 45, the thresholds correspond to 15 mm, 27 mm, and 14 mm, respectively.

The control group comprised female patients between the ages of 30 and 45 years who underwent classic abdominal dermolipectomy and did not present with diastasis recti according to the Rath criteria.

Exclusion criteria included previous abdominal surgery, abdominal hernia (diagnosed preoperatively or intraoperatively), collagen disease, diabetes mellitus, or corticosteroid use within 1 year of the study.

All patients underwent routine preoperative clinical and laboratory assessment for abdominal dermolipectomy and were deemed fit to undergo the procedure. Smokers were automatically deemed unfit to undergo dermolipectomy and were thus excluded from the sample.

### Procedures

#### Abdominal dermolipectomy and specimen collection

After preoperative markings and incision, all excess lower abdominal tissue was removed down to the aponeurotic layer. The remaining skin flap was undermined and raised, exposing muscle aponeuroses and the linea alba. Strict hemostasis was then achieved.

Upon exposure of the abdominal wall, the medial borders of both recti were identified and stained with methylene blue. The distance between the borders was measured 3.0 cm above the umbilicus and 2.0 cm below it. Depending on the measured distance, patients were diagnosed as having or not having diastasis and were then allocated to the case or control group accordingly.

Tissue samples (0.5×0.5 cm) were collected from the predefined supra- and infraumbilical measurement sites. The biopsy sites were sutured with 2-0 monofilament nylon. The surgery then proceeded as usual with plication of the linea alba. The skin flap was placed onto the abdominal wall and the umbilicus was reconstructed. Vacuum drains were placed, and skin closure was performed in a layered fashion.

#### Specimen processing

**Figure 1:**
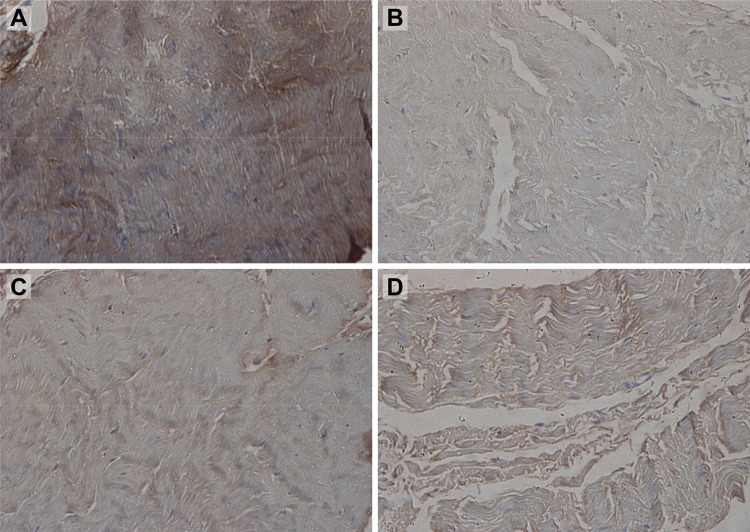
Photomicrographs of linea alba aponeurosis specimens stained using immunohistochemical methods for type I collagen (original magnification, 400×) and digitized using the application Image-Pro-Plus 3.1. The brown areas identify type I collagen. **A**: supraumbilical sample from patient with no diastasis; **B**: supraumbilical sample from patient with diastasis; **C**: infraumbilical sample from patient with no diastasis; **D**: infraumbilical sample from patient with diastasis.

Qualitative analysis of type I and type III collagen content was performed through immunohistochemistry using polyclonal anti-collagen type I and type III antibodies (Figures 1 e 2).

Ten fields of view on each slide (original magnification, 400×) were randomly digitized from a total of 1,440 images. Digitized slides were analyzed using the application Image Pro-Plus (Media Cybernetics, Silverspring, USA). Brown-stained (positive) areas were measured in each slide. To minimize bias and interference in the best possible way, the same researcher digitized and analyzed all images in the same workstation.

## DETERMINING THE STUDY SAMPLE SIZE

To obtain a significance level of α=0.05 and a statistical power of 80% and to detect a difference of one standard deviation in collagen type I and III levels between the groups with and without recti diastasis, we estimated that 18 cases were required in each group. 

### Statistical analysis

The data are expressed as the means and standard deviations.

Student’s *t*-test was used for initial comparisons. An analysis of covariance (ANCOVA) model was used to adjust for potential confounders (age and body mass index). For analysis of ratios, numerical values were log- and rank-transformed and applied to the ANCOVA model. The level of significance was set at α=0.05.

## RESULTS

Mean patient age was 39.6±4.4 years in the case (diastasis) group and 38.3±3.5 years in the control (no diastasis) group (Table 1). No nulliparous patients were present in either group.

As noted in Table 1, the distance between the medial borders of the recti was measured above and below the umbilicus in all patients as part of the allocation method. In the diastasis group, the mean distance between the recti was 3.21±0.94 cm in the supraumbilical region and 2.73±0.89 cm in the infraumbilical region. In the control group, the mean distance was 0.98±0.05 cm in the supraumbilical region and 0.89±0.03 cm in the infraumbilical region (Table 1).

Table 1 shows the results of quantitative assessment of type I and III collagen in the supra- and infraumbilical samples from both study groups. In controls (no diastasis), type I and III collagen were significantly more abundant in both supra- and infraumbilical specimens (*p*<0.001).

**Figure 3:**
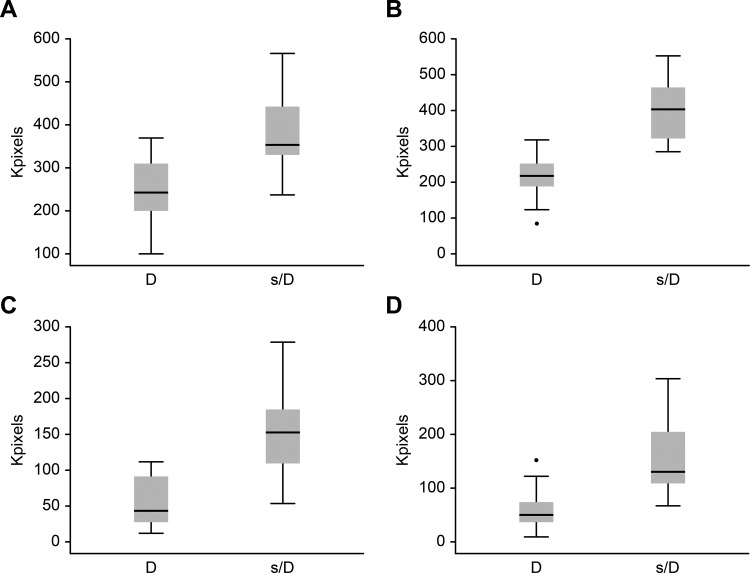
Box-and-whisker plots of differences between the diastasis recti (D) and no diastasis recti (ND) groups in the following variables: **A**: supraumbilical type I collagen; **B**: infraumbilical type I collagen; **C**: supraumbilical type III collagen; **D**: infraumbilical type III collagen.

Figure 3 shows box-and-whisker plots of the differences in supraumbilical type I collagen, infraumbilical type I collagen, supraumbilical type III collagen and infraumbilical type III collagen between the diastasis (D) and no diastasis (ND) groups.

## DISCUSSION

The present study sought to quantify and compare the amount of type I and III collagen in the linea alba of women with and without diastasis recti.

Many studies have focused on collagen, and the association between this protein and the etiology of hernias (due to its role in the weakening of aponeuroses) has been described extensively. Individuals with lower collagen content in aponeurotic structures have been found to be more prone to developing hernias 4,7-10.

Szczesny et al. studied the expression of type I and III collagen in the fascia of obese patients and observed a decrease in the relative expression level of type I and III collagen 5.

Lazarenko et al. studied collagen I and III levels in the skin and aponeuroses as a predictor of the risk of postoperative ventral hernia 11.

The observation of a wide heterogeneity in the linea alba of patients undergoing abdominal dermolipectomy and the countless studies that have demonstrated the importance of collagen in the structure and tensile strength of aponeuroses piqued our interest in quantitating total collagen and type I and III collagen content in this anatomical structure.

We found that patients without diastasis recti had higher levels of type I and type III collagen content in the linea alba than patients with diastasis, suggesting that the linea alba is less fragile in patients who do not develop diastasis.

Pregnancy is one of the various factors that predispose women to diastasis recti (widening of the linea alba) 12. However, a study by Hsia and Jones 13 found great postpartum variability in this anatomical structure, with some patients even exhibiting spontaneous resolution of diastasis. Our findings corroborate this information, because all patients in our sample—cases and controls alike—had previously given birth, which suggests that pregnancy *per se* cannot be a causative factor of permanent diastasis recti.

A review of the literature indicates that the distance between the medial borders of the recti is greater above the umbilicus than below 6,14-16. This finding was confirmed in our study. Only 1 out of 18 patients with diastasis recti had a distance between the medial borders of the recti that was greater in the infraumbilical region than in the supraumbilical region. In all controls (no diastasis), the distance between the medial borders of the recti in the supraumbilical region was equal to or greater than the distance measured below the umbilicus.

In both study groups, we found in absolute values proportionally more collagen type III in the infraumbilical region than in the supraumbilical region of the linea alba, which may suggest increased elasticity of the infraumbilical region of the abdomen, providing greater retractability and decreasing the distance between the medial borders of the recti. However, these values were not statistically significant.

The abdominal cavity is constantly exposed to fluctuations in intra-abdominal pressure, whether due to variation in intra-abdominal contents, contraction of the abdominal wall muscles, or movement of the thoracic cavity. The anterior abdominal wall, with particular assistance from its muscles and aponeuroses, counters these pressure increases. It is reasonable to presume that aponeurotic weakness along the midline would lead to abdominal protrusion; accordingly, diastasis recti is cited by Repta and Hunstad 17 as one of the causative factors of protrusion. However, Brauman 18 found that not all patients with bulging abdomens had diastasis recti, and *vice versa*. This finding should lead to reflection on the role of the muscle layer, and not only the aponeurotic layer, in providing resistance against intra-abdominal pressures.

In women with diastasis recti, type I and type III collagen—as measured in linea alba specimens collected both above and below the level of the umbilicus—are less abundant than in women without diastasis.

As in patients with hernias, women with diastasis recti present lower type I and III collagen levels in the studied fascia. The alterations reported in this work suggest consequences in the midline when type I and III collagens are reduced in this area.

## AUTHOR CONTRIBUTIONS

Blotta RM was responsible for the acquisition, analysis and interpretation of data, and manuscript drafting. Trindade EN was responsible for the critical revision of the manuscript. Costa SS was responsible for data acquisition. Meurer L was responsible for data analysis. Maciel-Trindade MR was responsible for study design and critical revision of the manuscript.

## Figures and Tables

**Figure 2:**
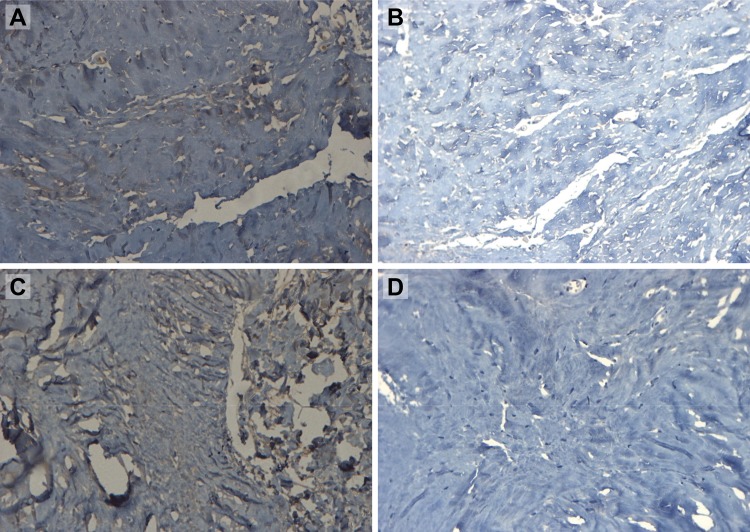
Photomicrographs of linea alba aponeurosis specimens stained using immunohistochemical methods for type III collagen (original magnification, 400×) and digitized using the Application Image-Pro-Plus 3.1. The brown areas identify type III collagen. **A**: supraumbilical sample from patient with no diastasis; **B**: supraumbilical sample from patient with diastasis; **C**: infraumbilical sample from patient with no diastasis; **D**: infraumbilical sample from patient with diastasis.

**Table 1:** Between-group comparison of select study variables.

Variable	Diastasis (n=18)	No diastasis (n=18)	*p*
Age (years)	39.6±4.4	38.3±3.5	0.323^a^
BMI (kg/m^2^)	25.0±1.5	24.3±1.5	0.127^a^
Distance between recti			
supraumbilical (cm)	3.21±0.94	0.98±0.05	<0.001^a^
infraumbilical (cm)	2.73±0.89	0.89±0.03	<0.001^a^
Type I collagen (SU), kpixels	244.5±73.5	381.1±101.1	<0.001^b^
Type I collagen (IU), kpixels	217.1±58.8	397.4±82.9	<0.001^b^
Type III collagen (SU), kpixels	54.3±33.1	154.9±59.4	<0.001^b^
Type III collagen (IU), kpixels	58.5±36.9	152.0±68.0	<0.001^b^
Hazard ratio C-III/C-I (SU)	0.23±0.13	0.44±0.23	<0.001^b^
Hazard ratio C-III/C-I (IU)	0.29±0.21	0.40±0.19	0.110^b^

BMI, body mass index, SU, supraumbilical; IU, infraumbilical; kpixels, pixels ×1000; C-III, type III collagen; C-I, type I collagen. *p*, *p-*value.

a Student’s *t*-test.

b analysis of covariance adjusting for potential effects of age and BMI.

## References

[1] Nahas FX, Barbosa MV, Ferreira LM (2009). Factors that may influence failure of the correction of the musculoaponeurotic deformities of the abdomen. Plast Reconstr Surg.

[2] Nahas FX, Ferreira LM, Ely PB, Ghelfond C (2011). Rectus diastasis corrected with absorbable suture: a long-term evaluation. Aesthetic Plast Surg.

[3] Henriksen NA, Mortensen JH, Sorensen LT, Bay-Jensen AC, Agren MS, Jorgensen LN (2015). The collagen turnover profile is altered in patients with inguinal and incisional hernia. Surgery.

[4] Fachinelli A, Maciel Trindade MR (2007). Qualitative and quantitative evaluation of total and types I and III collagens in patients with ventral hernias. Langenbecks Arch Surg.

[5] Szczesny W, Szczepanek J, Tretyn A, Dabrowiecki S, Szmytkowski J, Polak J (2015). An analysis of the expression of collagen I and III genes in the fascia of obese patients. J Surg Res.

[6] Rath AM, Attali P, Dumas JL, Goldlust D, Zhang J, Chevrel JP (1996). The abdominal linea alba: an anatomo-radiologic and biomechanical study. Surg Radiol Anat.

[7] Franz MG (2008). The biology of hernia formation. Surg Clin North Am.

[8] Henriksen NA, Yadete DH, Sorensen LT, Agren MS, Jorgensen LN (2011). Connective tissue alteration in abdominal wall hernia. Br J Surg.

[9] Rosch R, Junge K, Knops M, Lynen P, Klinge U, Schumpelick V (2003). Analysis of collagen-interacting proteins in patients with incisional hernias. Langenbecks Arch Surg.

[10] Wolwacz Junior I, Maciel-Trindade MR, Cerski CT (2003). [The collagen in transversalis fascia of direct inguinal hernia patients treated by videolaparoscopy]. Acta Cir Bras.

[11] Lazarenko VA, Ivanov SV, Ivanov IS, Rosberg EP, Tsukanov AV, Popova LP (2017). [Collagen types ratio in prediction of postoperative ventral hernias]. Khirurgiia (Mosk).

[12] Borges FS, Valentin EC (2002). Tratamento da flacidez e diástase do reto-abdominal no puerpério de parto normal com o uso de eletroestimulação muscular com corrente de média freqüência - estudo de caso. Rev Bras Fisioterapia Dermato-Funcional.

[13] Hsia M, Jones S (2000). Natural resolution of rectus abdominis diastasis. Two single case studies. Aust J Physiother.

[14] Beer GM, Schuster A, Seifert B, Manestar M, Mihic-Probst D, Weber SA (2009). The normal width of the linea alba in nulliparous women. Clin Anat.

[15] Nahas FX, Augusto SM, Ghelfond C (1997). Should diastasis recti be corrected. Aesthetic Plast Surg.

[16] Pontes R (2004). Abdominoplastia - ressecção em bloco e sua aplicação em lifting de coxa e torsoplastia.

[17] Repta R, Hunstad JP (2009). Diastasis recti: clinical anatomy. Plast Reconstr Surg.

[18] Brauman D (2008). Diastasis recti: clinical anatomy. Plast Reconstr Surg.

